# Incidence of stroke in the first year after diagnosis of cancer—A systematic review and meta-analysis

**DOI:** 10.3389/fneur.2022.966190

**Published:** 2022-09-20

**Authors:** Ronda Lun, Danielle Carole Roy, Yu Hao, Rishi Deka, Wen-Kuan Huang, Babak B. Navi, Deborah M. Siegal, Tim Ramsay, Dean Fergusson, Risa Shorr, Dar Dowlatshahi

**Affiliations:** ^1^Division of Neurology, Department of Medicine, The Ottawa Hospital Research Institute, Ottawa, ON, Canada; ^2^School of Epidemiology, University of Ottawa, Ottawa, ON, Canada; ^3^Department of Clinical Epidemiology, Ottawa Hospital Research Institute, Ottawa, ON, Canada; ^4^Biomedical Sciences Department, University of Calgary, Calgary, AB, Canada; ^5^Department of Psychiatry, University of California, San Diego School of Medicine, San Diego, San Diego, CA, United States; ^6^Department of Radiation Medicine and Applied Sciences, University of California, San Diego School of Medicine, San Diego, San Diego, CA, United States; ^7^Veterans Affairs Health Care System, San Diego, CA, United States; ^8^Division of Hematology/Oncology, Department of Internal Medicine, Chang Gung Memorial Hospital at Linkou, College of Medicine, Chang Gung University, Taoyuan, Taiwan; ^9^Clinical and Translational Neuroscience Unit, Brain and Mind Research Institute and Department of Neurology, Weill Cornell Medicine, New York, NY, United States; ^10^Department of Neurology, Memorial Sloan Kettering Cancer Center, New York, NY, United States; ^11^Division of Hematology, Department of Medicine, The Ottawa Hospital, Ottawa, ON, Canada; ^12^Department of Education, The Ottawa Hospital, Ottawa, ON, Canada

**Keywords:** stroke, cancer, population health, epidemiology, meta-analysis, systematic review, incidence, risk

## Abstract

**Background:**

Patients newly diagnosed with cancer represent a population at highest risk for stroke. The objective of this systematic review and meta-analysis was to estimate the incidence of stroke in the first year following a new diagnosis of cancer.

**Methods:**

We searched MEDLINE and EMBASE from January 1980 to June 2021 for observational studies that enrolled adults with a new diagnosis of all cancers excluding non-melanoma skin cancer, and that reported the incidence of stroke at 1 year. PRISMA guidelines for meta-analyses were followed. Two reviewers independently extracted data and appraised risk of bias. We used the Dersimonian and Laird random effects method to pool cumulative incidences after logit transformation, and reported pooled proportions as percentages. Statistical heterogeneity was assessed using the *I*^2^ statistic.

**Results:**

A total of 12,083 studies were screened; 41 studies were included for analysis. Data from 2,552,121 subjects with cancer were analyzed. The cumulative incidence of total stroke at 1 year was 1.4% (95% CI 0.9–2.2%), while the pooled incidence of ischemic stroke was 1.3% (95% CI 1.0–1.8%) and 0.3% (95% CI 0.1–0.9%) for spontaneous intracerebral hemorrhage (ICH), with consistently high statistical heterogeneity (>99% *I*^2^).

**Conclusion:**

The estimated incidence of stroke during the first year after a new diagnosis of cancer is 1.4%, with a higher risk for ischemic stroke than ICH. Cancer patients should be educated on the risk of stroke at the time of diagnosis. Future studies should evaluate optimal primary prevention strategies in this high-risk group of patients.

**Systematic review registration:**

https://osf.io/ucwy9/.

## Introduction

Cancer is a well-known risk factor for stroke (1), and both conditions are associated with a high degree of morbidity and mortality. There appears to be an increased risk for arterial thromboembolic events in the months preceding a diagnosis of cancer (2–4). Many studies have found that the risk for stroke increases around the time of cancer diagnosis and that this elevated risk may persist for up to 10 years after diagnosis (1). While this risk attenuates over time, it is hypothesized that the first year after a new diagnosis of cancer represents the period during which cancer patients have the highest stroke risk (2). From a clinical care standpoint, identifying patients with a new diagnosis of cancer who have not yet experienced a stroke represents an opportunity to study and implement primary prevention strategies in order to maximize quality of life in cancer patients. Thus, the objective of this systematic review and meta-analysis was to examine the risk of stroke in the first year following a new diagnosis of cancer.

## Methods

### Data availability statement

The data underlying this article will be shared on reasonable request to the corresponding author.

### Study protocol and registration

The protocol for this study was registered at the Open Science Framework (osf.io/ucwy9) and published in a peer-reviewed journal (1). This study was conducted based on the guidelines of the Cochrane Handbook of Systematic Reviews (2). It was reported using the updated guidelines for Preferred Reporting Items for Systematic Reviews (PRISMA) (3).

### Inclusion criteria

Our systematic literature search was limited to studies in adults with a new diagnosis of cancer, encompassing all cancer subtypes except non-melanoma skin cancers (i.e., basal cell and squamous cell carcinoma) due to their favorable prognosis and treatment with local measures not requiring systemic therapy, resulting in inaccuracies in administrative diagnostic coding. Studies must report the incidence of ischemic stroke and/or spontaneous intracerebral hemorrhage (ICH) during the first year after cancer diagnosis. If a graphical representation of the data was provided without a corresponding numerical value, the incidence at 1 year was extracted using the Engauge Digitizer software (4, 5). As the primary objective of this systematic review was to synthesize the natural history (i.e., incidence) of stroke in the cancer population, our search was limited to only observational studies and we excluded any interventional studies, as they represent a different population—it is estimated that <5% of adult cancer patients enroll in clinical trials (6), and this population is comparatively much healthier and younger than the general cancer population (7). Further details on our inclusion/exclusion criteria can be found in our published protocol (1).

### Search strategy

Our search strategy was conducted with the assistance of a health science librarian with expertise in systematic reviews (R.S.). We searched MEDLINE and EMBASE *via* OVID and PubMed and included all relevant studies from January 1980 to June 2021. A sample search strategy can be found in Supplementary Table 1; detailed search strategies can be found in our published protocol (1). We further searched the abstract databases from both the International Conference of Stroke and the European Stroke Organization Conference for the same time period. Our search was restricted to human adult subjects, and the language was limited to English language only.

### Article and data extraction

**Figure 1:**
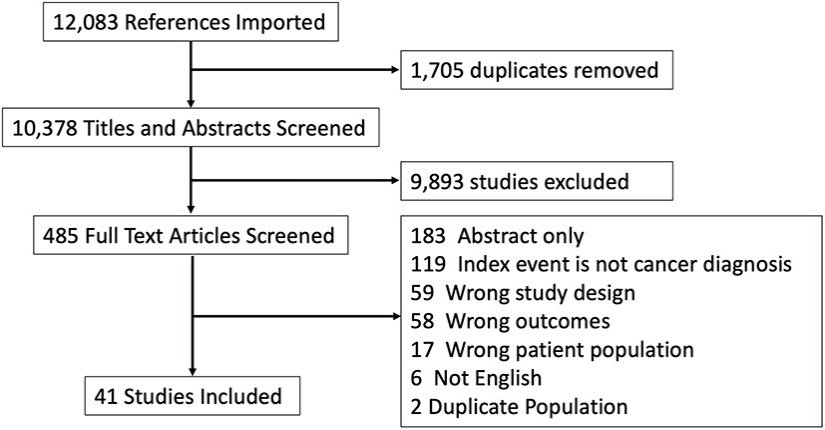
PRISMA flow diagram.

Two reviewers (R.L. and D.C.R.) independently completed two-level screening for articles using the Covidence Systematic Review software. Any discrepancies were resolved by discussion with a third senior author (D.D.). Findings from the screening process were summarized using a flow diagram (Figure 1).

A standardized data extraction form was created a priori and piloted independently by the two reviewers. The data extraction form was separated into bibliographic information [i.e., study ID, authors, title, funding status, journal, country and year of publication, study type, and name of databased used (if applicable)], subject information (i.e., number of participants total, number of participants with cancer, sex, age, cancer type, stage, and prevalence of comorbidities), and outcomes (i.e., incidence of ischemic stroke and/or ICH at 1 month, 3 months, 6 months, and 1 year). Wherever information had to be extracted using the Engauge Digitizer, only information at 1 year was extracted.

### Data collection process

One reviewer (R.L.) performed primary data extraction from the included studies and a second reviewer (D.C.R.) peer reviewed the extracted information. Disagreements were resolved *via* discussion. For studies that met inclusion criteria but did not report the specific outcome we required, the corresponding author of the study was contacted for the data a minimum of two times.

### Risk of bias assessment

Risk of bias (ROB) of individual studies was assessed using the Newcastle-Ottawa Scale (NOS) for cohort studies (8). Two raters (R.L. and D.C.R.) independently implemented the tool for all included studies and any disagreements in the rating were resolved by discussion. The following thresholds for converting the NOS to the Agency for Healthcare Research and Quality (AHRQ) standards are:

- Good quality: 3 or 4 stars in selection domain AND 1 or 2 stars in comparability domain AND 2 or 3 stars in outcome/exposure domain.
- Fair quality: 2 stars in selection domain AND 1 or 2 stars in comparability domain AND 2 or 3 stars in outcome/exposure domain.
- Poor quality: 0 or 1 star in selection domain OR 0 stars in comparability domain OR 0 or 1 stars in outcome/exposure domain.

### Statistical analysis

Our primary outcome was the cumulative incidence of stroke (total, ischemic, and hemorrhagic) at 1 year after a new diagnosis of cancer, and defined as the proportion of events divided by the sample size. Given the low number of studies that reported the incidence of stroke at 1, 3, and 6 months, we only reported the incidence of total stroke at these timepoints after pooling ischemic and hemorrhagic stroke. The transformed incidences were pooled using the Dersimonian and Laird random effects method (9). Between-study heterogeneity was assessed by the *I*^2^ statistic. To explore potential sources of heterogeneity, we caried out pre-specified subgroup analyses, including stratification based on ROB assessments, year of publication, location of primary cancer for studies that studied only one subtype of cancer, presence of atrial fibrillation, study design (i.e., prospective vs. retrospective cohorts), and nature of study (i.e., population-based or hospital-based). Univariate meta-regression analyses were carried out to test the influence of subgroup effects on heterogeneity. Sensitivity analyses were performed with the leave-one-out method to assess the influence of each study on the overall effect-size estimate. As this is a meta-analysis of pooled proportions from single cohorts (i.e., does not assess the effect of an intervention), publication bias was not assessed (2).

All statistical analyses were performed using the OpenMeta-Analyst software (10, 11).

## Results

Our search identified 12,083 studies across all databases searched. After removing duplicates, we screened 10,378 titles and abstracts and found 485 potentially eligible titles. During the second stage of full-text screening, we further excluded 444 studies (Figure 1), resulting in 41 full-text articles for data extraction and synthesis.

### Study selection and characteristics

The characteristics of included studies are listed in Table 1. The median year of publication was 2018 [interquartile range (IQR) 2015–2019]. Of the 41 studies, 18 were from Asia, 12 were from the United States, and 11 were from Europe. In total, they contributed 10,637,699 subjects for analysis, and 2,552,121 subjects had a new diagnosis of cancer. The sample size of cancer patients in the included studies ranged from 48 to 820,491, with a median sample size of 7,479 (IQR 893–22,737). Overall, 46% of the patients were women. Three studies were prospective cohort studies (12, 13), and the remaining were retrospective cohort studies. There were 29 studies that examined a single type of cancer (i.e., lung cancer), and the remaining studies included multiple types of cancer (Table 1).

**Table 1:** Included full-text articles for data extraction and meta-analysis.

**Study ID**	**First author**	**Title**	**Journal**	**Year**	**Study type**	**Cancer type enrolled**
1781	Andersen, Klaus Kaae	Risk of Ischemic and Hemorrhagic Strokes in Occult and Manifest Cancers	Stroke	2018	Retrospective case control	Multiple: 12.7% lung; 14.6% prostate, 2.3% CNS, 7.2% kidney and bladder, 3.9% urogynecologic, 2.7% pancreatic, 12.6% colorectal, 1.7% stomach, 5.1% melanoma, 2.5% head and neck, 3.1% myeloproliferative neoplasms
420	Bigelow, Elaine O	Burden of comorbidities is higher among elderly survivors of oropharyngeal cancer compared with controls.	Cancer	2020	Retrospective case control	Head and Neck
7128	Chan, Po-Chi	Higher stroke incidence in the patients with pancreatic cancer	Medicine	2018	Retrospective Cohort study	Pancreatic
2511	Chang, Wei-Chun	A nationwide population-based retrospective cohort study: decreased risk of stroke in cervical cancer patients after receiving treatment.	Archives of gynecology and obstetrics	2013	Retrospective Cohort study	Cervical
1319	Chen, Dong-Yi	Risk of Cardiovascular Ischemic Events After Surgical Castration and Gonadotropin-Releasing Hormone Agonist Therapy for Prostate Cancer: A Nationwide Cohort Study.	Journal of clinical oncology	2017	Retrospective Cohort study	Prostate
2077	Chu, C-N	Young nasopharyngeal cancer patients with radiotherapy and chemotherapy are most prone to ischaemic risk of stroke: a national database, controlled cohort study.	Clinical otolaryngology	2013	Retrospective case control	Head and Neck: 100% nasopharyngeal cancer
1265	Coutinho, Anna D	Elevated Cardiovascular Disease Risk in Patients With Chronic Myelogenous Leukemia Seen in Community-based Oncology Practices in the United States.	Clinical lymphoma, myeloma and leukemia	2017	Retrospective Cohort study	Hematologic: 100% chronic myelogenous leukemia
552	Deka, Rishi	Stroke and thromboembolic events in men with prostate cancer treated with definitive radiation therapy with or without androgen deprivation therapy.	Prostate cancer and prostatic diseases	2019	Retrospective Cohort study	Prostate
2837	Donato, Jessica	Intracranial hemorrhage in patients with brain metastases treated with therapeutic enoxaparin: a matched cohort study	Blood	2015	Matched Cohort	Central Nervous System: 100% brain metastases
2721	Du, Xianglin L	Risks of Venous Thromboembolism, Stroke, Heart Disease, and Myelodysplastic Syndrome Associated With Hematopoietic Growth Factors in a Large Population-Based Cohort of Patients With Colorectal Cancer.	Clinical colorectal cancer	2015	Retrospective Cohort study	Colorectal
1107	Du, Xianglin L	Associations between hematopoietic growth factors and risks of venous thromboembolism, stroke, ischemic heart disease and myelodysplastic syndrome: findings from a large population-based cohort of women with breast cancer.	Cancer causes and Control	2016	Retrospective Cohort study	Breast
4405	Geiger, Ann M	Stroke risk and tamoxifen therapy for breast cancer.	Journal of the National Cancer Institute	2004	Nested Case Control	Breast
6733	Gurnari, Carmelo	Early intracranial hemorrhages in acute promyelocytic leukemia: analysis of neuroradiological and clinico-biological parameters	British Journal of Hematology	2021	Retrospective Cohort study	Hematologic: 100% acute promyelocytic leukemia
2599	Hong, Julian C	Risk of cerebrovascular events in elderly patients after radiation therapy versus surgery for early-stage glottic cancer.	International Journal of Radiation Oncology, Biology, Physics	2013	Retrospective Cohort study	Head and Neck: 100% glottic cancer
6104	Navi, Babak	The risk of arterial thromboembolic events after cancer diagnosis	Research and Practice in Thrombosis and Hemostasis	2019	Prospective Cohort Study	Multiple: 21% prostate, 15% breast, 13% unknown primary, 11% lung, 8% colorectal, 5% bladder, 3% for leukemia, non-Hodgkin lymphoma and melanoma, 2% for kidney, head and neck, ovarian and primary brain, 1% for pancreas, multiple myeloma, uterine, gastric, esophageal, liver, and thyroid
7839	Jang, Hyun-Soon	The long-term effect of cancer on incident stroke: A nationwide population-based cohort study in Korea	Frontiers in Neurology	2019	Retrospective Cohort study	Multiple: proportions of subtypes not specified
845	Khosrow-Khavar, Farzin	Aromatase Inhibitors and the Risk of Cardiovascular Outcomes in Women With Breast Cancer: A Population-Based Cohort Study.	Circulation	2020	Retrospective Cohort study	Breast
6859	Kim, Do Kyung	Does androgen-deprivation therapy increase the risk of ischemic cardiovascular and cerebrovascular diseases in patients with prostate cancer? A nationwide population-based cohort study	Journal of Cancer Research and Clinical Oncology	2021	Retrospective Cohort study	Prostate
6222	Kim, Kyeong Jin	Effects of radioactive iodine treatment on cardiovascular disease in thyroid cancer patients: a nationwide cohort study	Annals of Translational Medicine	2020	Retrospective Cohort study	Thyroid
7134	Kim, Kyu	Effect of non-Vitamin K antagonist oral anticoagulants in atrial fibrillation patients with newly diagnosed cancer	Korean Circulation Journal	2018	Retrospective Cohort study	Multiple: proportions of subtypes not specified
600	Kitano, Takaya	The Effect of Chemotherapy on Stroke Risk in Cancer Patients.	Thrombosis and Haemostasis	2020	Retrospective Cohort study	Multiple: 12.9% breast, 10.9% uterus, 8.9% gastric, 7.8% for prostate, 7.8% for colorectal, 7.3% lung, 6.3% esophageal, 4.8% oropharyngeal, 4.2% hepatic, 3.3% pancreatic, 21.5% others, 4.4% hematopoietic cancers
2832	Kuan, Ai-Seon	Risk of Ischemic Stroke in Patients With Gastric Cancer: A Nationwide Population-Based Cohort Study.	Medicine	2015	Retrospective Cohort study	Gastric
2255	Kuan, Ai-Seon; Teng	Risk of ischemic stroke in patients with ovarian cancer: a nationwide population-based study	BMC Medicine	2014	Retrospective Cohort study	Ovarian
6476	Kwon, Hyun-Keun	The incidence of myocardial infarction and stroke in head and neck cancer patients	Scientific Reports	2021	Retrospective Cohort study	Head and Neck
1550	Lee, Gin-Yi	Risk of stroke in patients with newly diagnosed multiple myeloma: a retrospective cohort study	Hematological oncology	2017	Retrospective Cohort study	Hematologic
10377	Libourel, Eduard J	High incidence of arterial thrombosis in young patients treated for multiple myeloma: Results of a prospective cohort study	CLINICAL TRIALS AND OBSERVATIONS	2009	Prospective Cohort Study	Hematologic
6895	Liu, Peter Pin-Sung	High 1-year risk of stroke in patients with hepatocellular carcinoma: a nationwide registry-based cohort study	Scientific Reports	2021	Retrospective Cohort study	Hepatic
1379	Mantia, Charlene	Predicting the higher rate of intracranial hemorrhage in glioma patients receiving therapeutic enoxaparin.	Blood	2017	Matched Retrospective Cohort Study	Central Nervous System
6920	Mulder, FI, Nick	Arterial Thromboembolism in Cancer Patients: A Danish Population-Based Cohort Study	JACC: CardioOncology	2021	Retrospective Matched Cohort study	Multiple: 18.7% breast, 16.4% lung, 16.9%, 14.9% prostate, colorectal, 9.3% hematologic, 7.0% gynecological, 4.0% esophageal/stomach, 3.5% pancreatic, 3.5% bladder, 2.7% renal, 1.9% brain, 1.3% hepatic
1750	Navi, Babak B	New diagnosis of cancer and the risk of subsequent cerebrovascular events.	Neurology	2018	Prospective Cohort Study	Multiple: proportions of subtypes not specified
2397	Navi, Babak B	Association between incident cancer and subsequent stroke.	Annals of Neurology	2015	Retrospective matched cohort study	Multiple: 24.7% lung, 21.2% breast, 29.0% prostate, 5.1% pancreatic, 20.1% colorectal
151	Pardo Sanz, Ana	Current status of anticoagulation in patients with breast cancer and atrial fibrillation.	Breast	2019	Retrospective Cohort study	Breast
460	Plaja, Andrea	Thromboembolism and bleeding in patients with cancer and mechanical heart valves.	Journal of Thrombosis and Thrombolysis	2019	Retrospective Matched Cohort study	Multiple: 22% urogynecologic, 21% gastrointestinal, 21% other, 10% lung, 12% breast, 15% hematologic
7240	Szepligeti, Szimonetta	Vascular diseases in patients with chronic myeloproliferative neoplasms—Impact of comorbidity	Clinical Epidemiology	2019	Retrospective matched cohort study	Hematologic
554	Toulis, Konstantinos A	Risk of incident circulatory disease in patients treated for differentiated thyroid carcinoma with no history of cardiovascular disease.	Clinical Endocrinology	2019	Retrospective Matched Cohort Study	Thyroid
2479	Tsai, Shiang-Jiun	Increased risk of ischemic stroke in cervical cancer patients: a nationwide population-based study.	Radiation Oncology	2013	Retrospective Cohort study	Cervical
2569	van Herk-Sukel, Myrthe P P	Pulmonary embolism, myocardial infarction, and ischemic stroke in lung cancer patients: results from a longitudinal study.	Lung	2013	Retrospective Matched Cohort study	Lung
7874	Wu, Chia-Lun	Stroke rate increases around the time of cancer diagnosis	Frontiers in Neurology	2019	Retrospective Cohort study	Multiple: 15.0% lung, 15.0% colorectal, 13.0% hepatic, 7.5% urogenital, 6.7% gastric, 6.5% prostate
506	Yasui, Taku	Oral Anticoagulants in Japanese Patients with Atrial Fibrillation and Active Cancer.	Internal Medicine	2019	Retrospective Cohort Study	Multiple: 44.2% gastrointestinal, 24.1% lung, 11.2% urogynecologic, 9.8% head and neck, 4.0% breast, 3.1% hematologic, 3.6% others
1254	Zhang, Qiaolei	Risk factors and clinical characteristics of non-promyelocytic acute myeloid leukemia of intracerebral hemorrhage: A single center study in China.	Journal of Clinical Neuroscience	2017	Retrospective Cohort study	Hematologic
3788	Zoller, Bengt	Risk of haemorrhagic and ischaemic stroke in patients with cancer: a nationwide follow-up study from Sweden.	European Journal of Cancer	2012	Retrospective Cohort study	Multiple: proportions of subtypes not specified

### Total stroke (ischemic stroke and ICH) after cancer diagnosis

**Figure 2:**
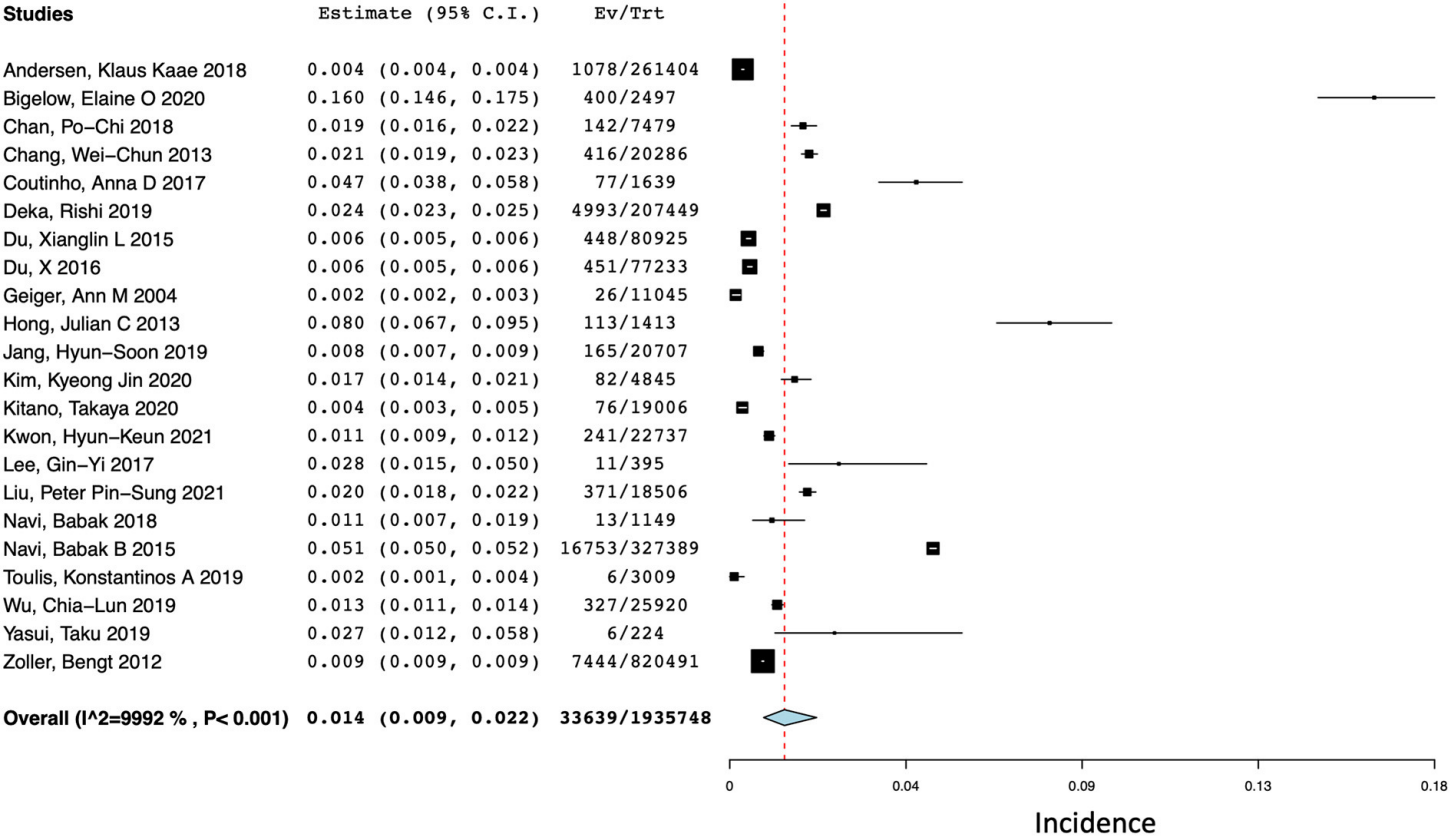
Forest plot of the pooled incidence of total stroke (ischemic and hemorrhagic) at 1 year after a diagnosis of cancer.

Of the included 41 studies, 22 reported the incidence of combined ischemic stroke and spontaneous ICH at 1 year (12, 14–34). For studies that reported the incidence of ischemic stroke and ICH separately, total stroke events were calculated by the sum of events. As shown in Figure 2, the pooled incidence of ischemic stroke and ICH at 1 year was 1.4% (95% CI 0.9–2.2%) with a high degree of statistical heterogeneity present (*I*^2^ = 99.92%).

Due to the low number of studies that reported the incidence of ischemic stroke and ICH at 1, 3, and 6 months, we only evaluated the cumulative incidence of total stroke (i.e., sum of events) at these timepoints. Five studies reported the incidence of stroke at 1 month (12, 16, 35–37), four reported the incidence of stroke at 3 months (12, 31, 35, 36), and eight reported the incidence of stroke at 6 months (12, 14, 16, 17, 31, 35, 36, 38). Their respective pooled incidences at 1, 3, and 6 months are 1.6% (95% CI 0.2–10.8%; *I*^2^ = 99.78%), 1.0% (95% CI 0.3–2.8%; *I*^2^ = 99.95%), and 1.3% (95% CI 0.6–2.9%; *I*^2^ = 99.96%) (Supplementary Figure 1).

### Ischemic stroke at one year after cancer diagnosis

**Figure 3:**
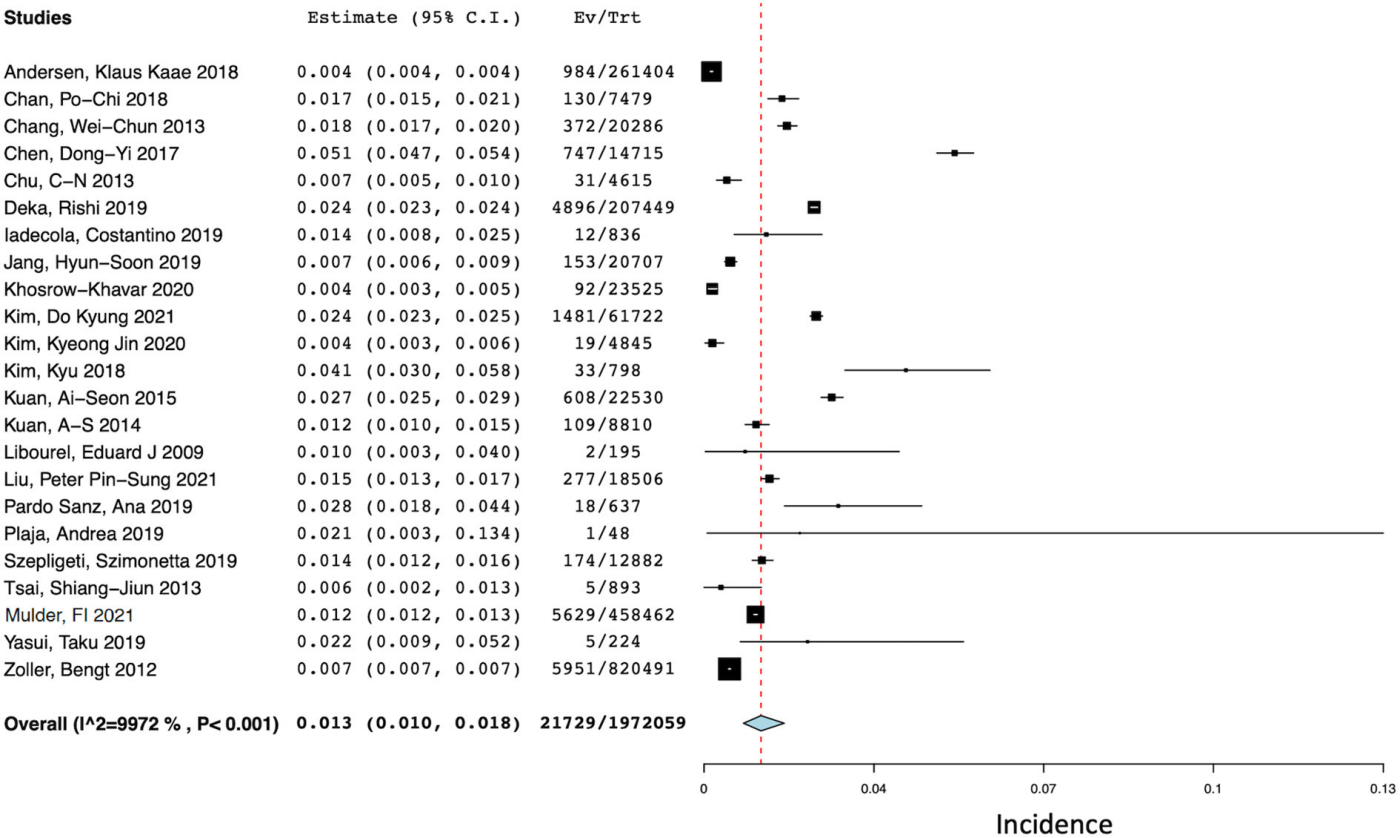
Forest plot of the pooled incidence of ischemic stroke at 1 year after a diagnosis of cancer.

Of the 41 studies, 23 (*N* = 1,972,059) reported the incidence of ischemic stroke events at 1 year after a diagnosis of cancer (13–15, 17, 18, 20, 25, 26, 30, 34–36, 39–49). The pooled incidence of ischemic stroke events during the first year after a new diagnosis of cancer (see Figure 3) was 1.3% (95% CI 1.0–1.8%) with a high degree of statistical heterogeneity present (*I*^2^ = 99.72%).

### Intracerebral hemorrhage at one year after cancer diagnosis

**Figure 4:**
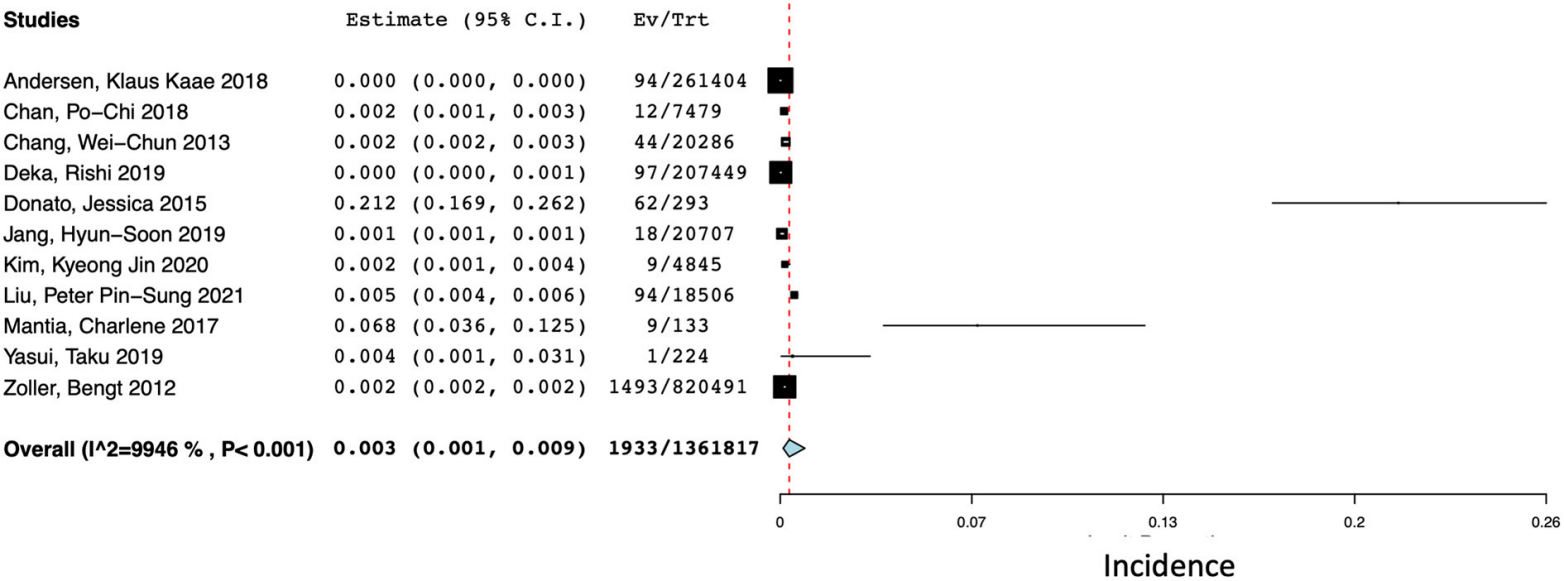
Forest plot of the pooled incidence of spontaneous ICH at 1 year after a diagnosis of cancer.

There were 11 studies (*N* = 1,361,817) that reported the incidence of ICH at 1 year after a diagnosis of cancer (14, 15, 17, 18, 20, 25, 26, 30, 34, 50, 51). The pooled incidences of ICH at 1 year after diagnosis was 0.3% (95% CI 0.1–0.9%) with a high degree of statistical heterogeneity present (*I*^2^ = 99.46%) (Figure 4).

### Subgroup analyses

#### Atrial fibrillation and ischemic stroke

Three studies exclusively included cancer patients with atrial fibrillation (34, 43, 46). Two of these studies specifically examined the use of anticoagulation in these patients (34, 43). while the third study reported approximately that 85% of their cohort was on anticoagulation (46). In a subgroup analysis of studies that only included cancer patients with atrial fibrillation, the incidence of ischemic stroke was 3.3% at 1 year (95% CI 2.4–4.6%) with relatively low heterogeneity (*I*^2^ = 29.70%), which was significantly higher than the incidence of ischemic stroke in 20 other studies [1.2% (95% CI 0.9–1.6%), *I*^2^ = 99.76%] (Supplementary Figure 2).

#### Cancer subtype

Out of 22 studies that reported the combined incidence of ischemic and hemorrhagic stroke at 1 year, eight were cohort studies that included multiple types of cancer (Supplementary Figure 3) (12, 14, 15, 25, 27, 31, 33, 34). The remaining 14 studies were limited to specific cancer subtypes including head and neck cancer (*n* = 3) (16, 24, 28), hematologic malignancies (*n* = 2) (19, 29), breast cancer (*n* = 2) (21, 23), and thyroid cancer (*n* = 2) (26, 32). There was only one study for each of the following subtypes of cancer: pancreatic, cervical, prostate, colorectal, and hepatic (17, 18, 20, 22, 30). Subgroup analyses based on cancer type found that the pooled incidence of stroke for the eight studies that enrolled multiple types of cancer had a similar incidence to our overall pooled incidence: 1.1% (95% CI 0.5–2.7%), with significant heterogeneity (*I*^2^ = 99.97%). Studies that enrolled head and neck cancer patients exclusively appeared to have a higher incidence of stroke [5.3% (95% CI 0.8–27.5%, *I*^2^ = 99.83%)], although this difference was not statistically significant. Studies that enrolled hematologic cancer patients also reported a higher incidence of stroke: 3.9% (95% CI 2.4–6.3%), with moderate heterogeneity, *I*^2^ = 63.64% (Supplementary Figure 3). Thyroid cancer appeared to have a non-significant lower risk of stroke: 0.6% (95% CI 0.1–4.8%), while breast cancer patients reported a lower risk of stroke that was statistically significant: 0.4% (95% CI 0.2–0.9%, *I*^2^ = 95.10%). Based on results from a single study each, it appeared that pancreatic cancer, cervical cancer, prostate cancer, and hepatic cancer all had elevated risk of stroke compared to the overall pooled incidence of stroke (Supplementary Figure 3).

#### Study design

Out of the 22 studies that reported 1-year incidence of total stroke, there were 5/22 that used a hospital-based sample, and 17/22 that used a population-based sample. There was no statistical difference between the two groups in terms of the overall incidence: 1.6% (95% CI 0.9–2.8%) for population-based studies and 1.4% (95% CI 0.9–2.2%) for hospital-based studies (Supplementary Figure 7). We also performed subgroup analyses stratified by retrospective vs. prospective design and did not find a significant difference in 1-year incidence between these two groups (Supplementary Figure 8): 1.5% (95% CI 0.9–2.3%) for retrospective studies and 1.4 (95% CI 0.7–1.9%) for prospective. It is also important to note that in the analysis for total stroke, only 1/22 studies were prospective and the remaining 21 were retrospective.

### Sensitivity analyses and meta-regression

Sensitivity analyses were performed with the leave-one-out method to examine the stability of the results (Supplementary Figure 4). We found that no single study significantly altered the pooled effect measure—cumulative incidences ranged from 1.3% (95% CI 0.8–2.0%) to 1.6% (95% CI 0.010–0.025), which was not statistically different from the pooled estimate of 1.4% (95% CI 0.9–2.2%). Subgroup analysis based on the geographic region of enrolled patients found that the risk of stroke appeared to be highest in cohorts from the United States (4.1%, 95% CI 2.8–5.3%) compared to cohorts from Asia (1.5%, 95% CI 1.8–2.7%) or Europe (0.5%, 95% CI 0.1–0.9%) (Supplementary Figure 5). Between-study heterogeneity was statistically quantified with univariate meta-regression analyses, which identified that substantial heterogeneity originated from differences in cancer type (*p* = 0.014), but not from year of publication, risk of bias, presence of atrial fibrillation, or study design (i.e., retrospective vs. prospective).

### Risk of bias in studies

Using the NOS risk of bias tool, we found that 6/41 studies were “poor quality,” one was “fair quality,” and the remaining 34 were “good quality” (Supplementary Table 2). Subgroup analysis stratified by the ROB ratings found that 20 of 22 studies that reported 1-year stroke outcomes were deemed “good quality” (12, 14–28, 30–33), while one received a “poor” rating (34) and one received a “fair” rating (29). The studies that received a “good” rating had a similar pooled incidence of stroke at 1 year compared to the primary analysis (Supplementary Figure 6): 1.4% (95% CI 0.9–2.2%, *I*^2^ = 99.92%). The study with a “poor” rating reported a slightly higher cumulative incidence of stroke at 1 year [2.7% (95% CI 1.2–5.8%)], although this difference was not statistically significant. The single study that received a “fair” rating similarly reported a slightly higher incidence of stroke at 1 year: 2.8% (95% CI 1.5–5.0%).

## Discussion

### Summary of findings

Through our systematic literature search, we identified that the cumulative incidence of any stroke during the first year after a new diagnosis of cancer was 1.4%. The risk of ischemic stroke was similar (1.3%), but the risk for spontaneous ICH was much lower comparatively, at 0.3%. The risk for ischemic stroke was particularly high in patients with cancer and atrial fibrillation, and in those with head and neck cancer and hematologic cancers. For context, the incidence of stroke in those aged 55 years or older has been reported to be ~5.3 per 1,000 person-years, which translates to a cumulative incidence of ~0.53% per year for a single individual (52). This suggests that compared to the general population, there is approximately a 2.6-fold increase in the risk for stroke during the first year after a new cancer diagnosis.

### Interpretation in the context of existing literature

There are two previously published systematic reviews that examined the risk of stroke in cancer survivors compared to cancer-free populations (53, 54). Zhang et al. found that the relative risk for stroke was 1.66 times higher (95% CI 1.35–2.04) for cancer survivors compared to the cancer-free population over an unspecified follow-up period (53). Turner et al. reported a hazard ratio of 1.22 (95% CI 1.12–1.33) across all stroke subtypes when examining adult cancer survivors. The slight differences between their estimates and ours is likely a reflection of differences in study population, follow-up time, and statistical pooling methods –by evaluating cancer *survivors*, both studies would have excluded malignancies with poor prognoses, and may have also excluded those who suffered a stroke already with consequent mortality. Zhang et al. also included those with childhood cancers and used relative risk to approximate and pool all effect measures (i.e., relative risk, hazard ratio, incidence rate ratio, standardized incidence ratios), which may have reduced accuracy, particularly when dealing with relatively rare outcomes (53). Additionally, our population likely represents one that is at highest risk for stroke, given the risk for stroke in cancer patients is not constant over time—previous studies have found that stroke risk increases during the months leading up to a diagnosis of cancer, and peaks around the time of diagnosis (55, 56). Turner et al. similarly reported that the incidence of stroke may be highest immediately after a diagnosis of new cancer (i.e., first 6 months) (54).

### Subgroup analysis: Geographic location

Our meta-analysis identified that the combined risk of stroke varied by geographic location: the risk of stroke was significantly higher in cohorts from the United States compared to the Asia and Europe. This is different than previously reported numbers of stroke risk by geographic region in the general population: East Asia has been reported to have the highest lifetime risk of stroke, followed by Central and Eastern Europe (57). The discrepancy reported in our study may reflect the heterogeneity that is present in baseline patient characteristics and cancer subtypes across studies. Using visual inspection of the forest plot, it is evident that the higher incidence of stroke in the United States cohort may be largely driven by three studies: Bigelow (study ID 420), Hong (study ID 2599) and B. Navi (study ID: 2397) (Supplementary Figure 5) (16, 24, 31). Two of these studies (Hong and Bigelow) were specifically investigating the risk of stroke in elderly patients with glottic and oropharyngeal cancer—the respective median/mean ages of cancer subjects include in these two cohort studies were 72 (IQR 69–77) and 73.8 (no standard deviation reported) (16, 24). The population from the 2015 study by Navi selectively included patients with breast, colorectal, lung, pancreatic, or prostate cancer from the Surveillance Epidemiology and End-Results (SEER) Medicare database—the authors chose these cancer types *a priori* because pancreatic cancer is the cancer type most commonly reported in association with thromboembolic events, and the remaining four represent the most common cancers reported in the United States population (31). This may therefore represent a highly selected group of patients, as gastric, lung and pancreatic cancers are particularly associated with a higher risk for stroke (53, 54, 58). These cancers are also more likely to be at advanced stages at the time of diagnosis, which is an independent risk factor to increase the risk for thromboembolic events (31). Unfortunately information regarding cancer stage was not available for this meta-analysis. Furthermore, the SEER-Medicare database restricts the study population to patients 65 and older so the higher rates of stroke may be partly driven by the older population—this is supported by the high rates of stroke seen in the matched control patients without cancer (31).

### Subgroup analysis: Atrial fibrillation

Our subgroup analysis found that in patients with cancer and atrial fibrillation, there was an elevated risk for ischemic stroke compared to the general cancer cohort: 3.3 vs. 1.3%, respectively. This is similar to what is reported in the literature—a large national registry-based cohort study reported that the annual incidence rate of ischemic stroke was 3.44% in those with atrial fibrillation and active or history of cancer (59). This risk is stratified by the presence of comorbidities, including older age, and the presence of other vascular risk factors (60, 61). While current risk stratification tools for atrial fibrillation (i.e., CHADS2 or CHADS2-VASC) do not take cancer into account, when applied in a population of cancer patients with atrial fibrillation, they were still found to be predictive of ischemic stroke (62). There is a higher prevalence of atrial fibrillation in patients with cancer compared to those without cancer—a relationship that persists regardless of surgery or cancer therapy (63). The mechanism by which this occurs is postulated to be multifactorial, but may be related to higher incidences of post-operative atrial fibrillation, chemotherapy-related adverse effects, cancer-associated systemic inflammation which promotes atrial re-structuring, and autonomic dysregulation in patients with malignancy (63–66). Thus, the co-occurrence of atrial fibrillation and cancer represents a highly susceptible population with the highest risk for ischemic stroke; these patients may require anticoagulation for primary prevention.

There are no randomized studies that evaluate the efficacy and safety of antithrombotic therapies specifically for the primary prevention of stroke in patients with new/active cancer. Initiation of antithrombotics in this population is challenging for multiple reasons. First, currently there is no risk stratification tool for accurately identifying patients with cancer at the highest risk for stroke. This represents the biggest challenge, as patients with cancer on antithrombotic therapy are also at increased risk for major bleeding compared to non-cancer patients, and therefore accurate patient selection is crucial (67). Second, the choice of antithrombotic for primary prevention should be highly individualized. For example, anticoagulation is recommended for the prevention of stroke in patients with atrial fibrillation, and it is generally accepted that direct oral anticoagulants (DOAC) are preferred to warfarin (68). While patients with cancer were excluded from the large DOAC trials comparing rivaroxaban, apixaban, dabigatran, and edoxaban to warfarin (69–72), recent observational data suggests that DOAC is comparable to warfarin in terms of efficacy and safety profiles, and the Canadian Cardiovascular Society recommends the use of DOAC as first line anticoagulation therapy in patients with atrial fibrillation and active cancer [weak recommendation; low-quality evidence] (68). However, many DOACs are mainly metabolized *via* cytochrome P450 3A4 in the liver, and concomitant use of DOACs with drugs that regulate this pathway may be contraindicated, as many chemotherapeutic agents fall in this category (73). This example highlights that the choice of antithrombotic therapy in cancer patients should be highly individualized due to multiple patient and treatment considerations, and therefore should be chosen under the guidance of an expert multidisciplinary team (68).

In contrast, there is high-level evidence to support the use of reduced-dose DOACs in the setting of primary prevention for venous thromboembolism (VTE) in ambulatory cancer patients starting chemotherapy at intermediate to high risk for VTE (defined as Khorana Score ≥2) (74, 75). In two large randomized controlled trials, reduced-dose apixaban and rivaroxaban lowered the risk for VTE compared to placebo with an acceptable risk of bleeding (74, 75). Although this indirect evidence generally supports the use of antithrombotic treatments to prevent cardiovascular events among newly diagnosed cancer patients, the low number of cerebrovascular events in both trials may limit the generalizability of these results (74, 75).

### Study heterogeneity

Our study reported a very high *I*^2^ value, indicative of substantial between-study heterogeneity. Significant heterogeneity is a well-recognized statistical phenomenon for meta-analyses of single proportions, and interpretation of *I*^2^ values in this context is challenging (53, 76). Extremely large sample sizes of single cohort studies result in very narrow confidence intervals, thereby *I*^2^ for pooled estimates can be extremely high even in the presence of modest inconsistency between studies (77). Moreover, despite the extreme values of heterogeneity, we feel these results are still valid and clinically informative, as the primary objective of this study was to provide a global estimate of the risk for stroke in newly diagnosed cancer patients—a heterogeneous population to begin with. Therefore, it may be reasonable to sacrifice statistical homogeneity for broad inclusion criteria encompassing different cancer subtypes, treatments, and settings to increase the generalizability of our results and statistical power (78). Using univariate meta-regression analyses, we found that substantial heterogeneity could be attributed to differences in cancer type, which is known to influence the risk of stroke (15). Residual heterogeneity likely also originates from differences in baseline patient characteristics which cannot be quantified in an aggregate-level meta-analysis (i.e., patient age, sex, presence of vascular comorbidities, differences in cancer treatment, and use of antithrombotics). For example, Pardo Sanz et al. examined a cohort of patients with breast cancer and atrial fibrillation, where 84.9% of patients were anticoagulated (46). However, as not everyone in the study was anticoagulated, this study could not be included in our subgroup analysis looking at the use of anticoagulation. Therefore, significant heterogeneity remains despite our attempts at quantification of the sources of heterogeneity.

Our meta-analysis provides significant clinical implications for the risk of stroke in patients newly diagnosed with cancer. Our study confirms that the risk of ischemic stroke is high compared to the general population during the first year after a cancer diagnosis, and these patients should be considered for primary prevention strategies. At present, there isn't enough evidence to support the use of antithrombotic therapy empirically in these patients. However, patients should at least be educated that their stroke risk is high during this time period, and patient education regarding healthy lifestyle habits (i.e., healthy diet, regular exercise, smoking cessation, alcohol consumption reduction) should be provided. Furthermore, patient education around what to look for in terms of signs or symptoms of stroke should also be emphasized to minimize delays in access to acute treatments.

### Strengths and limitations

Our study has several strengths to note. This meta-analysis produced one of the largest pooled cohorts of newly diagnosed patients with cancer in which to characterize the risk of stroke. We believe our pooled results will provide patients and clinicians with an accurate estimation of the cumulative incidence of stroke during a high-risk period. Our statistical methodology was robust, with the publication of an a priori, peer-reviewed study protocol and a comprehensive search strategy encompassing multiple databases (1). However, our study is not without limitations. First, our pooled results reported significant heterogeneity among the included studies, which is likely due to differences in baseline patient characteristics (i.e., cancer subtype, age, sex, presence of vascular risk factors, ethnicity, treatment and prognoses), as discussed above. However, we believe that the increased statistical power we report due to our large sample size is also a strength of our study and may result in increased generalizability of these results. Unfortunately due to the lack of access to individual patient data and significant variations in how age was summarized and reported across studies, we could not perform subgroup analyses stratified by age. Next, our meta-analysis only included observational cohort studies; we may have missed potential data from randomized/interventional studies and gray literature. This was intentional during the design of our study protocol, as we wanted to focus on the natural history of disease so the results are applicable to all cancer patients, and the clinical trial population is often highly selected in oncology literature. As this meta-analysis was conducted exclusively in the English language, we may have missed literature published in other languages, which might have introduced language bias, though previous studies found little to no effect on summary measures excluding non-English studies (79). Lastly, medical surveillance bias should be considered when interpreting studies reporting risk for other diseases in the cancer population, as cancer patients may be followed more closely than non-cancer patients given the complexities of treatment.

## Conclusion

We found that the estimated cumulative incidence of stroke during the first year after a new diagnosis of cancer is 1.4%, and the risk for ischemic stroke is higher than the risk for spontaneous ICH. Patients newly diagnosed with cancer require education around the risk of stroke at the time of their diagnosis, as well as signs and symptoms of stroke to watch out for to minimize delays in access to acute treatments. Healthcare providers should advocate for conservative primary prevention strategies in this group of high-risk patients.

## Data availability statement

The raw data supporting the conclusions of this article will be made available by the authors, without undue reservation.

## Author contributions

RL: conceptualization, data curation, formal analysis, investigation, methodology, software, writing—original draft, reviewing, and editing. DR: data curation, investigation, methodology, reviewing, and editing. YH, RD, W-KH, and BN: data curation, investigation, reviewing, and editing. DS: conceptualization, methodology, supervision, reviewing, and editing. TR: conceptualization, data curation, methodology, supervision, reviewing, and editing. DF: data curation, formal analysis, investigation, methodology, resources, software, supervision, and reviewing and editing. RS: data curation, methodology, and reviewing and editing. DD: conceptualization, data curation, formal analysis, investigation, methodology, resources, supervision, and reviewing and editing. All authors contributed to the article and approved the submitted version.

## Conflict of interest

The authors declare that the research was conducted in the absence of any commercial or financial relationships that could be construed as a potential conflict of interest. The reviewer Y-CW declared a shared affiliation with the author W-KH to the handling editor at the time of review.

## Publisher's note

All claims expressed in this article are solely those of the authors and do not necessarily represent those of their affiliated organizations, or those of the publisher, the editors and the reviewers. Any product that may be evaluated in this article, or claim that may be made by its manufacturer, is not guaranteed or endorsed by the publisher.
