# Supplementary material for: Incidence of stroke in the first year after diagnosis of cancer—A systematic review and meta-analysis
Source: Front Neurol. 2022 Sep 20;13:966190. doi: 10.3389/fneur.2022.966190 (PMC9530058; doi:10.3389/fneur.2022.966190)

**Incidence of Stroke in the First Year After Diagnosis of Cancer – A Systematic Review and Meta-Analysis: Supplemental Materials**

**Total number of Tables: 2**

**Total number of Figures: 8**

**eTable 1.** Search strategy using EMBASE via the OVID interface:

| 1     malignant neoplasm/ or neoplasm/ or solid malignant neoplasm/ or myeloproliferative neoplasm/ (567513) |
| --- |
| 2     (cancer or tumo?r* or neoplasms* or carcinoma or leuk?em* or myeloma* or melanoma or hodgkin* or lymphoma or malignanc* or oncology).tw. (5041168) |
| 3     1 or 2 (5128557) |
| 4     exp *brain hemorrhage/ (59384) |
| 5     *cerebrovascular accident/ (84430) |
| 6     brain ischemia/ (150108) |
| 7     ((brain or cerebr*) adj3 (isch?em* or h?emorrhag* or bleed*)).tw. (94262) |
| 8 stroke.ti. (167370) |
| 9 ((isch?em* or h?emorrhag*) adj3 stroke*).tw. (117459) |
| 10 cerebr* vascular accident*.tw. (2269) |
| 11     or/4-10 (379118) |
| 12     3 and 11 (17887) |
| 13     incidence/ (442103) |
| 14     incidence.tw. (1188620) |
| 15     risk*.tw. (3427729) |
| 16     *risk/ or *risk factor/ (150822) |
| 17     13 or 14 or 15 or 16 (4322259) |
| 18     12 and 17 (5883) |

**eTable 2.** Risk of bias assessments using the Newcastle-Ottawa Scale for the included articles for meta-analysis.

| **Study ID** | **First Author** | **Year** | **ROB Domain Selection** | **ROB Domain**  **Comparability** | **ROB Domain Outcome** | **ROB**  **Overall** |
| --- | --- | --- | --- | --- | --- | --- |
| 1781 | Andersen | 2018 | 3 | 2 | 3 | Good |
| 420 | Bigelow | 2020 | 3 | 2 | 2 | Good |
| 7128 | Chan | 2018 | 4 | 2 | 2 | Good |
| 2511 | Chang | 2013 | 4 | 2 | 2 | Good |
| 1319 | Chen | 2017 | 3 | 2 | 2 | Good |
| 2077 | Chu | 2013 | 4 | 2 | 2 | Good |
| 1265 | Coutinho | 2017 | 3 | 2 | 2 | Good |
| 552 | Deka | 2019 | 3 | 2 | 2 | Good |
| 2837 | Donato | 2015 | 3 | 2 | 3 | Good |
| 2721 | Du | 2015 | 4 | 2 | 3 | Good |
| 1107 | Du | 2016 | 4 | 2 | 3 | Good |
| 4405 | Geiger | 2004 | 3 | 2 | 2 | Good |
| 6733 | Gurnari | 2021 | 2 | 0 | 2 | Poor |
| 2599 | Hong | 2013 | 4 | 2 | 2 | Good |
| 6104 | Iadecola | 2019 | 4 | 2 | 3 | Good |
| 7839 | Jang | 2019 | 4 | 2 | 2 | Good |
| 845 | Khosrow-Khavar | 2020 | 3 | 2 | 3 | Good |
| 6859 | Kim | 2021 | 4 | 2 | 2 | Good |
| 6222 | Kim | 2020 | 3 | 2 | 3 | Good |
| 7134 | Kim | 2018 | 3 | 2 | 3 | Good |
| 600 | Kitano | 2020 | 3 | 2 | 3 | Good |
| 2832 | Kuan | 2015 | 4 | 2 | 3 | Good |
| 2255 | Kuan | 2014 | 4 | 2 | 3 | Good |
| 6476 | Kwon | 2021 | 4 | 2 | 2 | Good |
| 1550 | Lee | 2017 | 2 | 2 | 1 | Fair |
| 10377 | Libourel | 2009 | 3 | 2 | 2 | Good |
| 6895 | Liu | 2021 | 4 | 2 | 2 | Good |
| 1379 | Mantia | 2017 | 3 | 2 | 2 | Good |
| 1750 | Navi | 2018 | 3 | 2 | 2 | Good |
| 2397 | Navi | 2015 | 4 | 2 | 2 | Good |
| 151 | Pardo Sanz | 2019 | 2 | 2 | 1 | Poor |
| 460 | Plaja | 2019 | 3 | 2 | 1 | Poor |
| 7240 | Szepligeti | 2019 | 3 | 2 | 3 | Good |
| 554 | Toulis | 2019 | 4 | 2 | 2 | Good |
| 2479 | Tsai | 2013 | 4 | 2 | 2 | Good |
| 6920 | Mulder | 2021 | 3 | 2 | 1 | Poor |
| 2569 | van Herk-Sukel | 2013 | 4 | 2 | 2 | Good |
| 7874 | Wu | 2019 | 4 | 2 | 2 | Good |
| 506 | Yasui | 2019 | 3 | 2 | 1 | Poor |
| 1254 | Zhang | 2017 | 2 | 2 | 1 | Poor |
| 3788 | Zoller | 2012 | 3 | 2 | 2 | Good |

**eFigure 1.** Pooled incidence of total strokes (ischemic and intracerebral hemorrhage) at 1 month (a), 3 months (b), and 6 months (c).

**
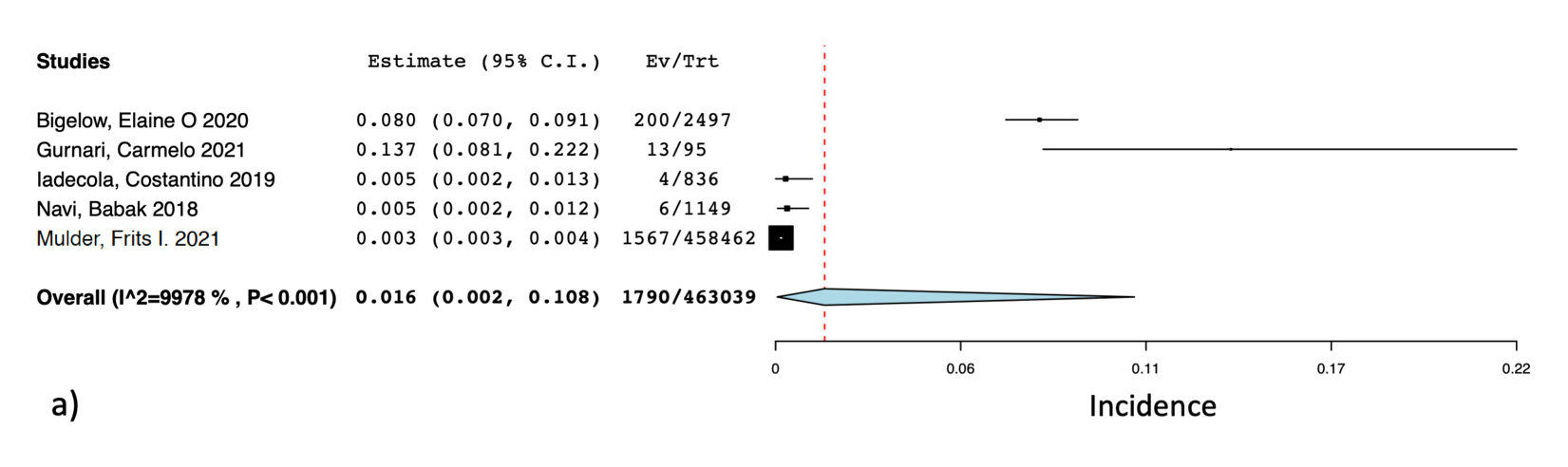
**

**
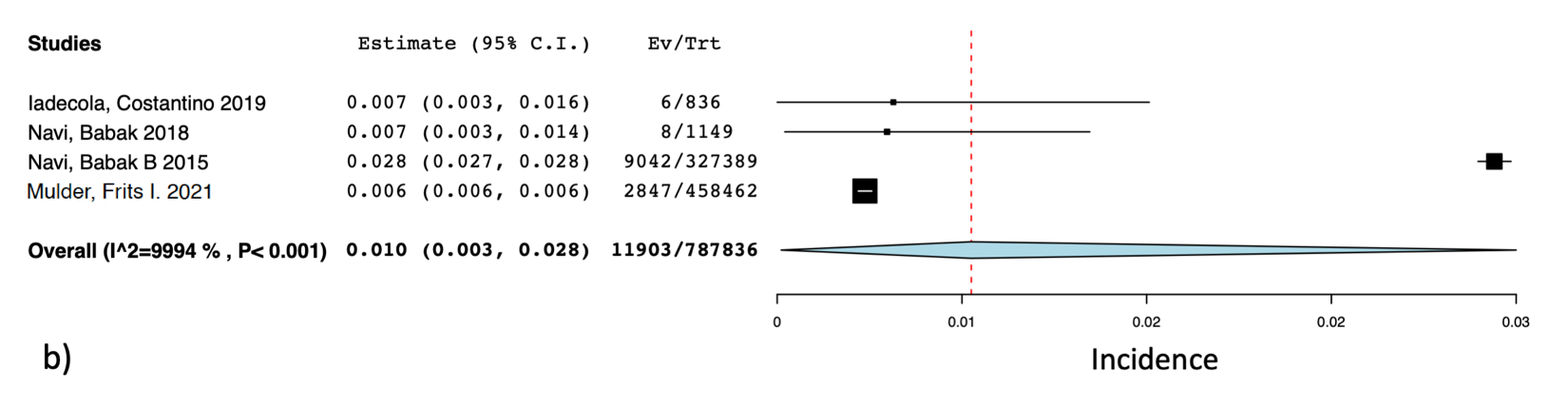
**

**
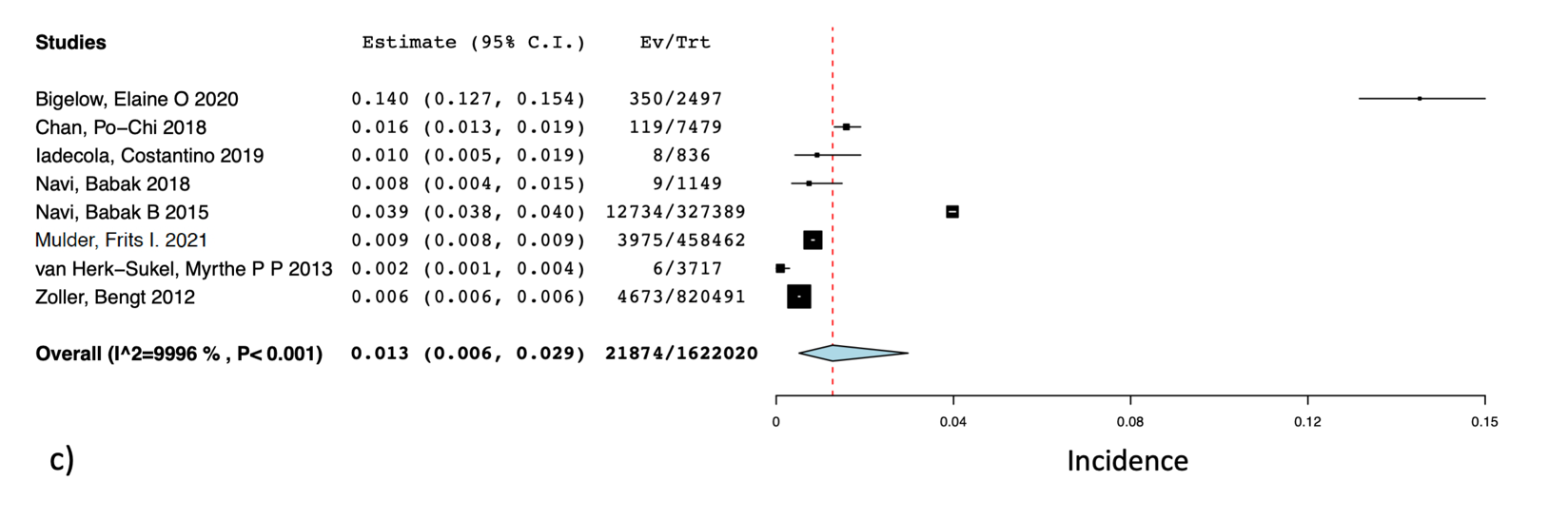
**

**eFigure 2.** Subgroup analysis of the incidence of ischemic stroke at 1 year, stratified by exclusive enrollment of cancer patients with atrial fibrillation.


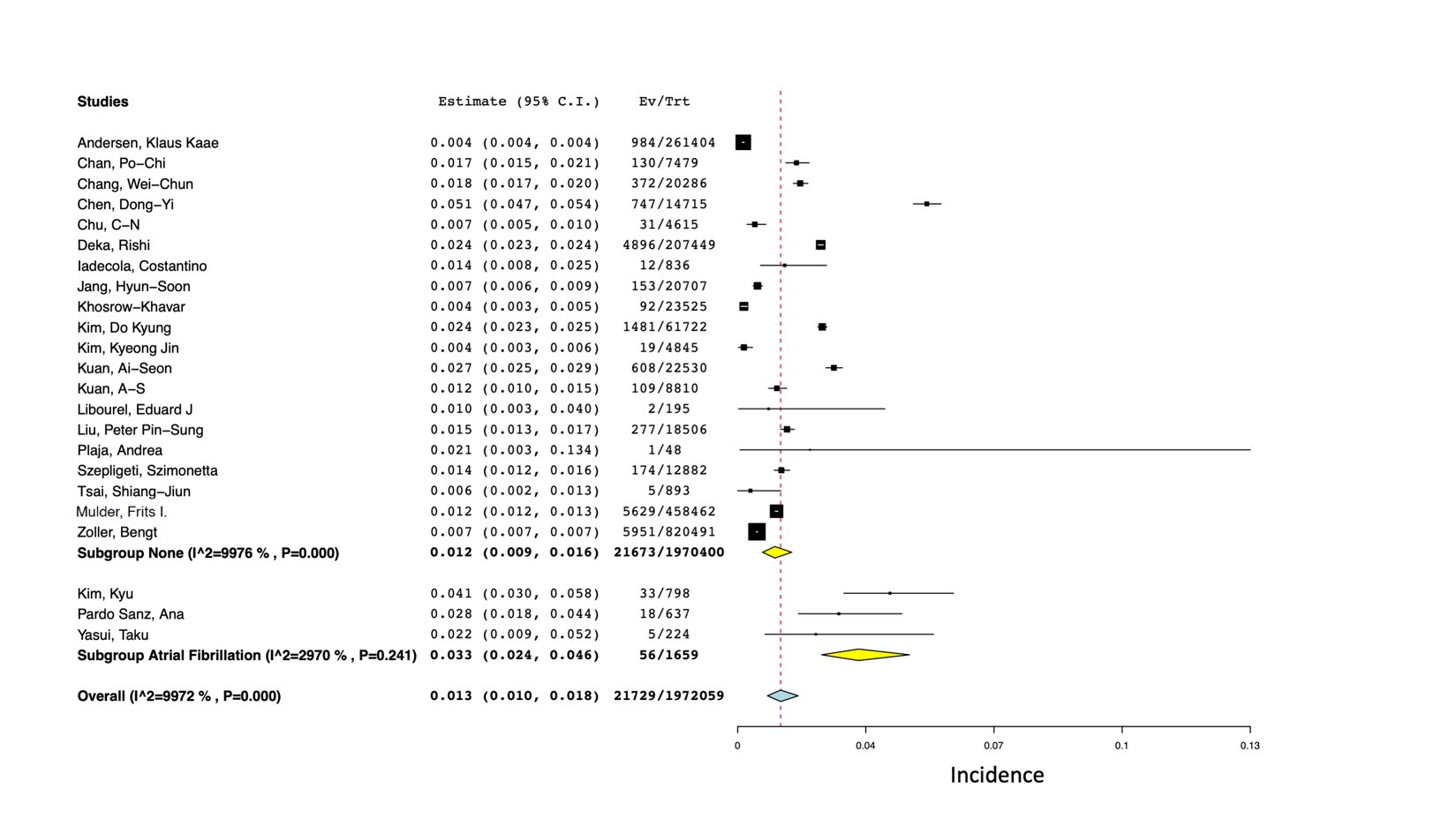


**eFigure 3**. Subgroup analysis of the incidence of total stroke at 1 year, stratified by cancer subtype.


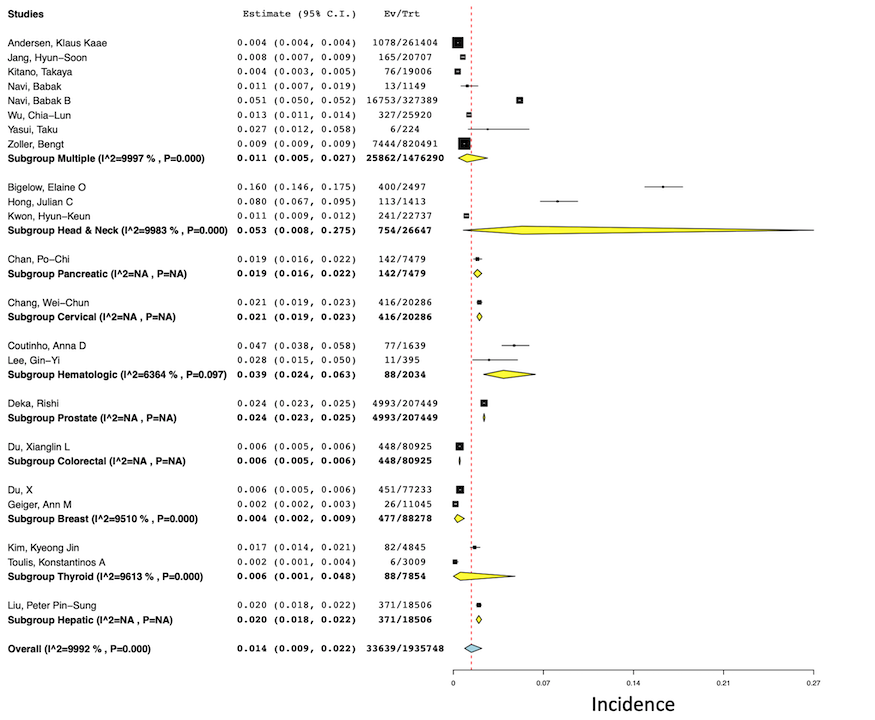


**eFigure 4.** Sensitivity analysis with leave-one-out method. Outcome assessed is the cumulative incidence of ischemic and hemorrhagic stroke.


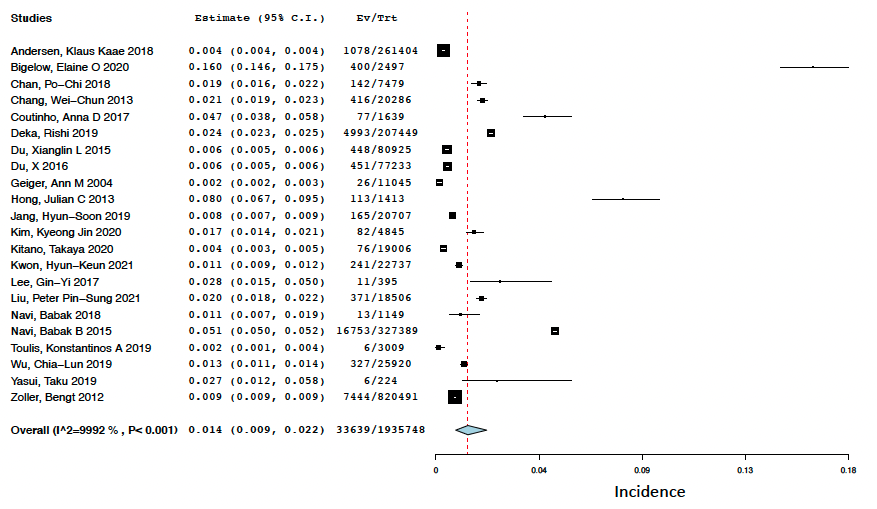


**eFigure 5.** Subgroup analysis of the risk for stroke (ischemic and hemorrhagic) based on geographic location.


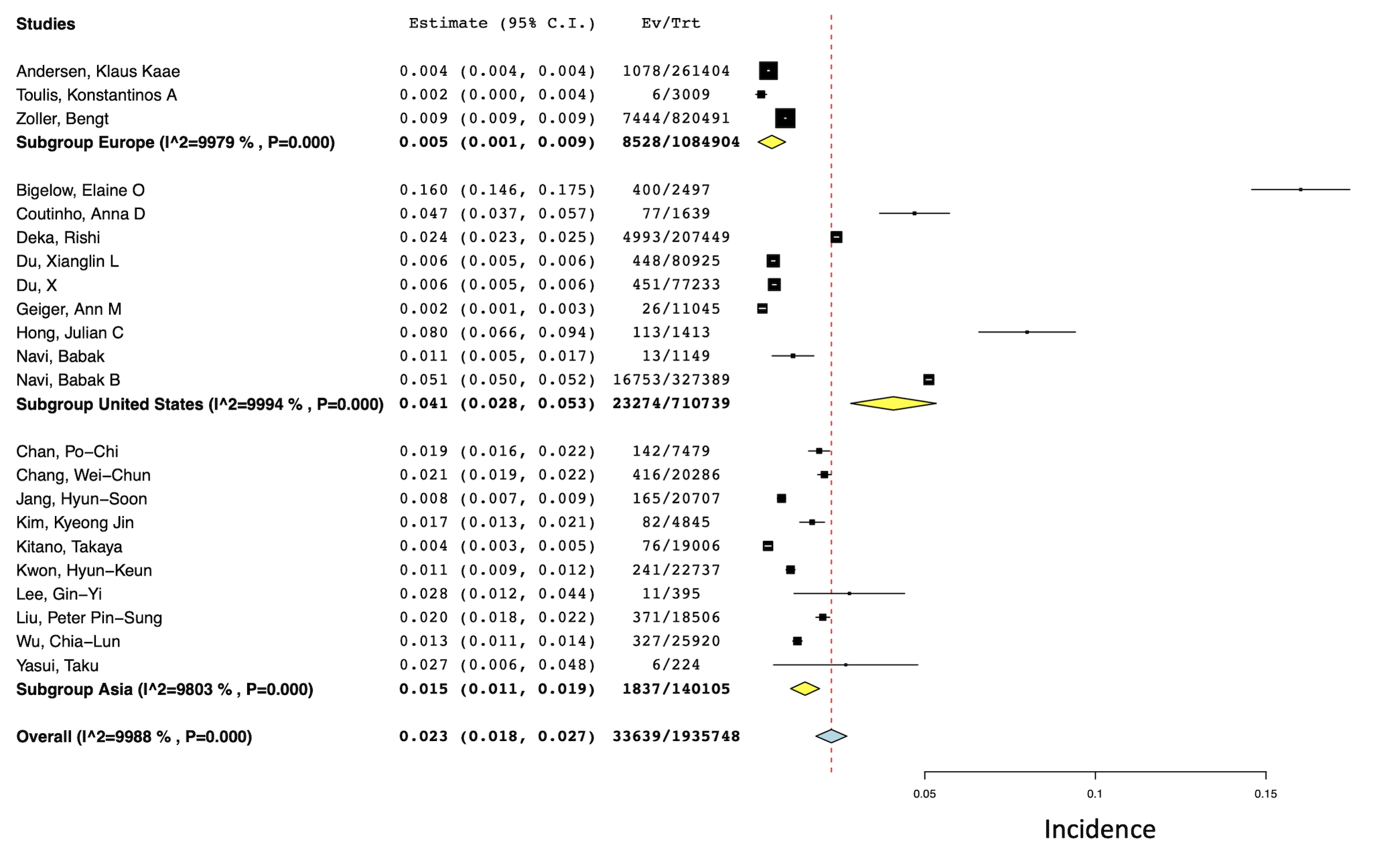


**eFigure 6**. Subgroup analysis stratified by risk of bias assessments using the Newcastle Ottawa Scale.


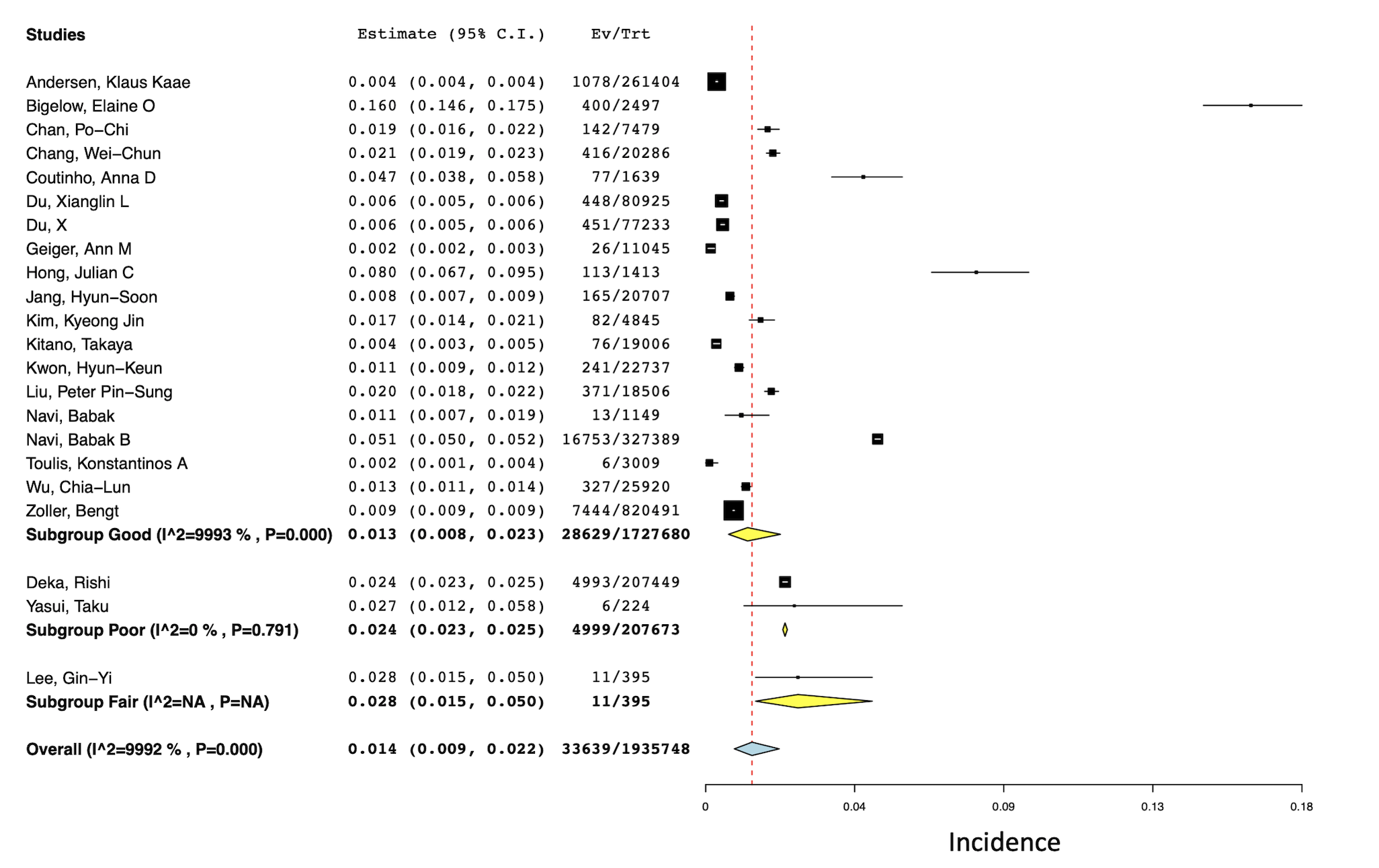


**eFigure 7**. Subgroup analysis stratified by nature of population studied: hospital-based or population-based.

**eFigure 8.** Subgroup analysis stratified by study design: retrospective vs prospective.


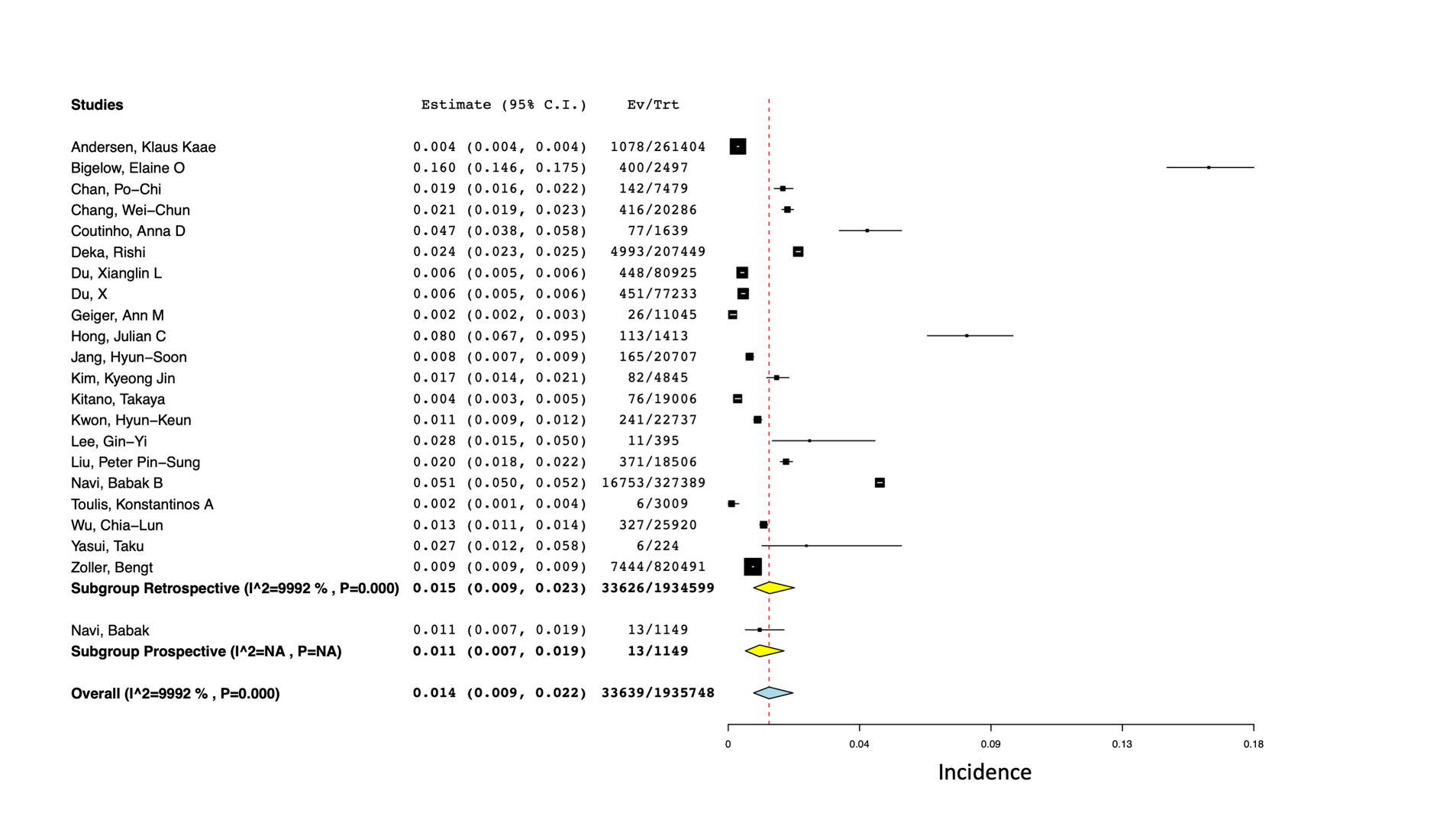

Supplement: Supplementary file 1 [file Table_1.docx]
